# Supplementary material for: Achieving High-Precision, Low-Cost Microfluidic Chip Fabrication with Flexible PCB Technology
Source: Micromachines (Basel). 2024 Mar 22;15(4):425. doi: 10.3390/mi15040425 (PMC11051900; doi:10.3390/mi15040425)
Supplement: Supplementary file 1 [file micromachines-15-00425-s001.zip › micromachines-2893046-supplementary.pdf]

Low Cost flexible PCB patterning for high accuracy Glass-PDMS Microfluidic chips

Supplementary documentation

Materials and Methods

2. Benchmark Design

Table S1. Overview of PCB features selected for each type

	Rigid FR-4 PCB	Flexible PCB
**Copper Thickness / Feature height**	1oz (35 µm) and 2oz (70µm)	1.5 oz (52.5 µm)
**Design Features**	Large copper pads at the beginning and end of MF channels are used to create an even and level bottom surface to be used during hole punching of PDMS before removing it from the master mold.	
	Double sided design	Single sided design
**Chip dimensions**	100x100 mm, this is the standard size when ordering a PCB, this results in a lower price.	75x100mm
**Usable surface area**	200 cm², enough to populate almost 11 standard 75x25mm microscope slides.	75 cm², enough to populate more than 4 standard 75x25mm microscope slides.
**Smallest feature size**	100 µm	60 µm
**Feature population**	Most features are laid out in a way to cut the PDMS casting in 75x25mm sections, and therefore simplifying the PDMS dicing process when later bonding the PDSM with standard microscope slides.	

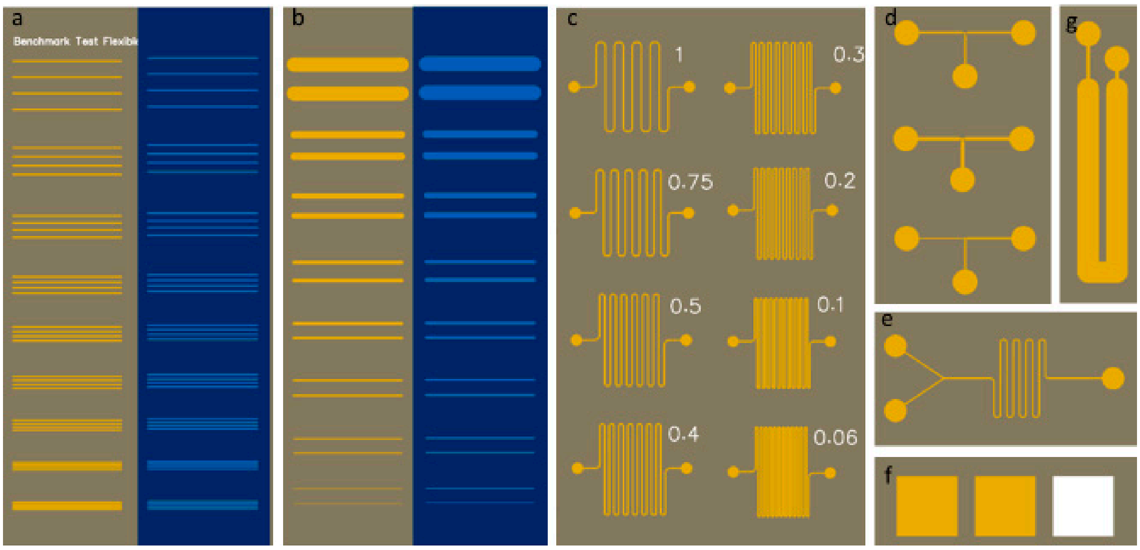

Fig. S1. Graphical presentation of several features included on the designs to test for performance with blue being covered in solder resist or cover lay and yellow not covered. Upper or lower limit of chosen dimensions are dependent on the type of PCB and their manufacturing limits. (a) Lines with a constant width, spaced closer together with separation of 1 mm down to 60 µm, (b) lines with 2 mm separation, ranging width from 2 mm down to 60 µm, (c) several serpentine channels to test the minimum spacing ranging from 1 mm down to 60 µm. (d) Several T-shaped droplet generator designs, (e) Flow merging design leading to a serpentine mixer, (f) Several regular surfaces to test surface quality of possible substrates and (g) an incubator channel allowing for the storage of larger volumes of liquid for an extended period of time.

3. Manufacturing

3.1. Design Specifications FR-4 vs Flex

Table S2. - Standard fabrication options compared between FR-4 and Flexible PCBs (Data from PCB Way, pcbway.com, last accessed 2023)

Feature	Capability (FR-4 PCB)	Capability (Flex PCB)
**Build Time**	3-5 days	3-5 days
**Base material**	FR-4, Aluminum	Polyimide Flexible (also PET)
**Thickness**	0.2-2.4mm	0.08-0.4mm
**Min Track/Spacing**	0.1mm/4mil	≥0.06mm/≥0.06mm
**Surface Finish**	HASL with lead<br>HASL lead free<br>Immersion gold, OSP	Immersion gold, OSP, Immersion silver, Immersion tin
**Finished Copper**	1oz-2oz-3oz (35µm-70µm-105µ)	0.5-2oz (16µm-70µm)

3.2. PDMS chip processing

Soft Lithography

The soft lithography process involves several meticulous steps to create microfluidic devices. Here is a more detailed description of the process:

Photoresin Application (a): The process begins with a silicon wafer that serves as the master mold. A layer of SU-8 photoresin is evenly applied to the silicon wafer through a technique known as spin coating. This results in a uniform layer of photoresin on the wafer. A mask is then employed to selectively shield parts of the photoresin layer from UV exposure. Using a UV curing light source, the exposed photoresin is solidified, while the unexposed areas remain uncured.

Development and Pattern Transfer (b): Following the exposure step, the wafer is subjected to a chemical development process. The uncured photoresin is carefully removed, leaving behind the desired pattern etched onto the silicon wafer. This pattern effectively forms the master mold that will define the microchannels and structures of the microfluidic device.

PDMS Molding (c): A two-component silicone, typically PDMS (Polydimethylsiloxane) mixed in a 10:1 ratio (commonly Sylgard 184 by Dow Corning), is prepared. This PDMS mixture is then poured onto the surface of the master mold created in the previous steps. After pouring, the PDMS is allowed to cure, often through baking or heating. Once cured, the PDMS adopts the negative imprint of the mold, effectively creating a replica of the desired microfluidic structure.

CWI Creation (d): To allow for the entry and exit of liquids into the microfluidic device, holes are created in the PDMS. These holes are often made using a biopsy punch and are strategically placed to form the Chip-to-World-Interface (CWI). The CWI is essential for connecting the device to external fluid sources or other components.

Surface Activation and Bonding (e): The open surface of the PDMS replica is treated with plasma. This plasma treatment activates the surface, making it more amenable to bonding with other materials. In this case, a glass microscope slide or cover slip is typically used. The plasma-activated PDMS and the glass substrate are brought into contact to form a tight seal. Care is taken to ensure no air bubbles are trapped between the two surfaces.

Final Bonding and Functional Chip (f): After the PDMS and glass have been joined, they are subjected to a short heat treatment. This heat treatment permanently bonds the PDMS to the glass, creating a fully functional microfluidic chip. The resulting device is now ready for use in various applications, including the controlled flow and manipulation of fluids at a microscale level.

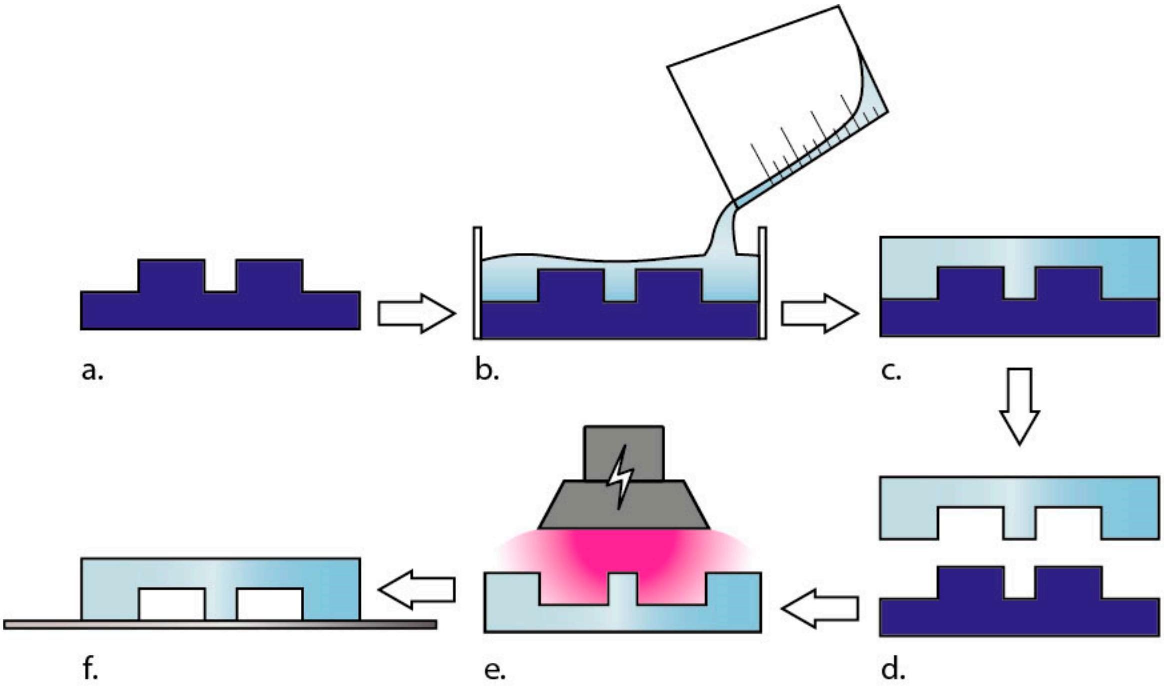

Fig. S2. Overview of the soft lithography process: (a) Selective patterning of photoresin, (b) Molding PDMS to create a negative replica, (c) Creation of Chip-to-World-Interface (CWI), (d) Plasma activation for surface bonding, (e) Careful bonding with a glass substrate, and (f) Completion of a fully functional microfluidic chip.

3.2.1. PDMS chip processing

Mold preparation

A flexible PCB was securely affixed to a porous MDF (Medium-Density Fiberboard) surface using a custom 3D-printed fixture. This fixture featured multiple thumbscrews located around the periphery of the PCB, ensuring a firm and stable attachment. To create an airtight seal along the edges, a gasket was placed between the 3D-printed frame and the MDF base plate. Additionally, the MDF base plate itself was made airtight through the application of aluminum tape.

To facilitate the vacuum process, a specially designed 3D-printed connector, produced with an SLA printer (Formlabs), was affixed to the lower part of the setup. This connector featured a silicone gasket to ensure a secure connection to the vacuum pump.

The flexible PCB was then subjected to tension by the vacuum, allowing for the casting of PDMS (Polydimethylsiloxane). The curing process occurred at room temperature over a span of two days, with the vacuum being maintained throughout the hardening process.

Additionally, an alternative and simpler method was subsequently discovered. In this method, the flexible PCB was delicately attached to a flat surface, specifically a 10 mm thick acrylic plastic sheet. To achieve this, double-sided pressure adhesive (Tesa SE, Germany) was used to securely bond the PCB to the acrylic surface, with great care taken to prevent the formation of any air pockets, which could compromise the quality of the mold.

To enclose the borders of the mold during the casting process, the previously mentioned painter's tape (3M, Minnesota, U.S.) was employed. This method exhibited superior efficiency and convenience compared to the previously described approach, particularly if executed without the introduction of air bubbles.

Design files of the 3D printed fixture can be downloaded in the digital supplement.

3.2.2. Mold Preparation Flexible PCB

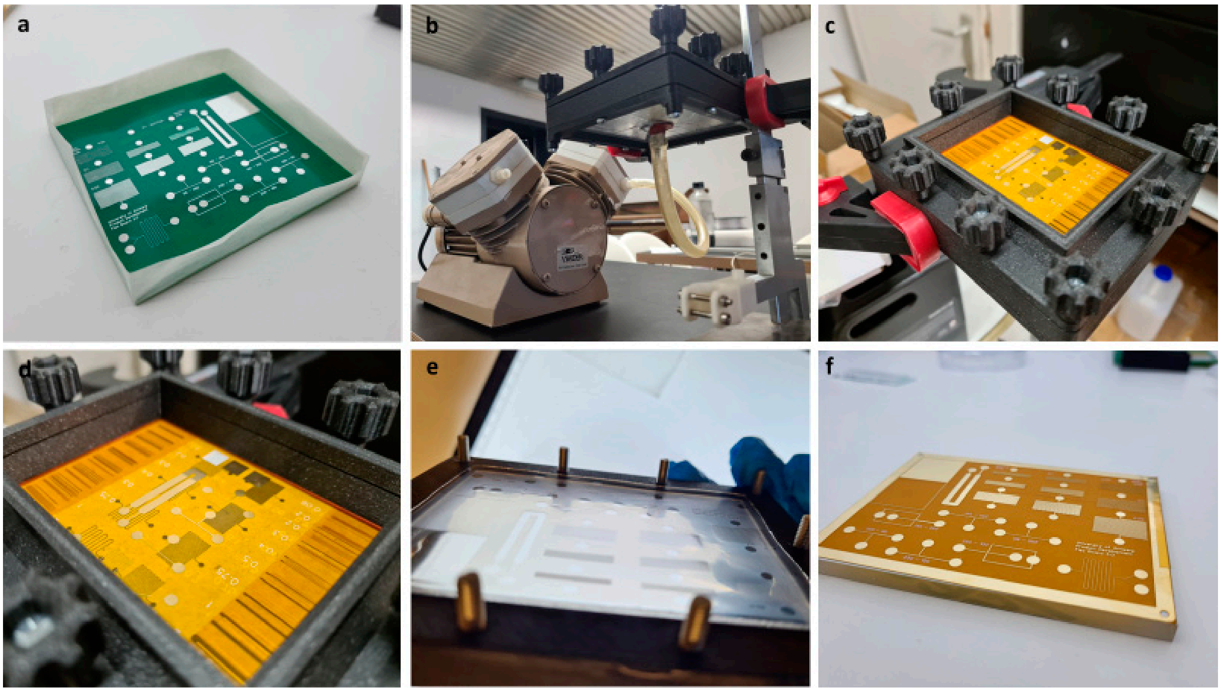

Fig. S3. This composite figure consists of six sequential images arranged with corresponding labels (a) to (f): (a) An FR-4 rigid PCB with its borders delineated by painter's tape. (b) A 3D-printed jig securely holds a flexible PCB, connected to a vacuum pump through a flexible PVC tube from the bottom of the setup. (c) An aerial view of the 3D-printed jig, offering a detailed perspective of the apparatus. (d) A close-up of the flexible PCB fixed within the 3D-printed jig, emphasizing the casting surface. (e) Depicts the PDMS casting process, with a photograph post-curing, revealing the final geometric shape of the cast PCB within the mold. (f) The flexible PCB is firmly affixed to an acrylic sheet, ensuring a flat and secure orientation during the casting process.

4. Cross Sectioning process

In order to enhance the visualization of the track geometry on printed circuit boards (PCBs), a more intricate inspection process was adopted, as conventional 2D-inspection methods were found inadequate for obtaining comprehensive insights. The following steps were performed as part of this process:

PCB Preparation (fig. S5. a-d): The PCBs were meticulously prepared for inspection. To facilitate detailed visualization, the boards were cut or sawn into smaller sections, which were then placed within custom-designed 3D printed containers.

Container Fabrication: The containers used to secure the PCB sections were crafted using PLA plastic filament through a Fused Deposition Modeling (FDM) printer, specifically the Prusa Mini from Prusa Research in Czechia.

Epoxy Encasement (fig. S5. d &g): To firmly encase the PCB sections and provide structural stability, a clear two-part epoxy, sourced from Resion in the Netherlands, was carefully cast to fill the interior of the containers. Once the epoxy was allowed to fully set and harden, the PCB sections were effectively encapsulated.

Precision Milling (fig. S5. e): The epoxy-encased PCB sections were then clamped within a machine vice and subjected to precision milling using a flat-end mill. This milling process continued until the depth of the desired features for inspection was achieved.

Surface Refinement (fig. S5. f & g): Subsequently, the top surface of the milled section underwent a meticulous refinement process. It began with sanding, gradually progressing from coarse (80 grit) to exceedingly fine (3000 grit)

sandpaper. Afterward, the surface was buffed using a microfiber cloth and a specialized buffing compound. This meticulous procedure resulted in a top surface that was not only smooth but also perfectly transparent, allowing for clear cross-sectional views of the PCB features.

Alignment for Inspection: The design of the PCB included channel lines on the side, facilitating easy alignment for insertion into the 3D printed container. This arrangement ensured that the PCB track faced the top surface perpendicularly, optimizing the inspection process for track geometry and related attributes.

By implementing this detailed procedure, a comprehensive and visually clear representation of the PCB's track geometry was achieved, enabling in-depth analysis and examination.

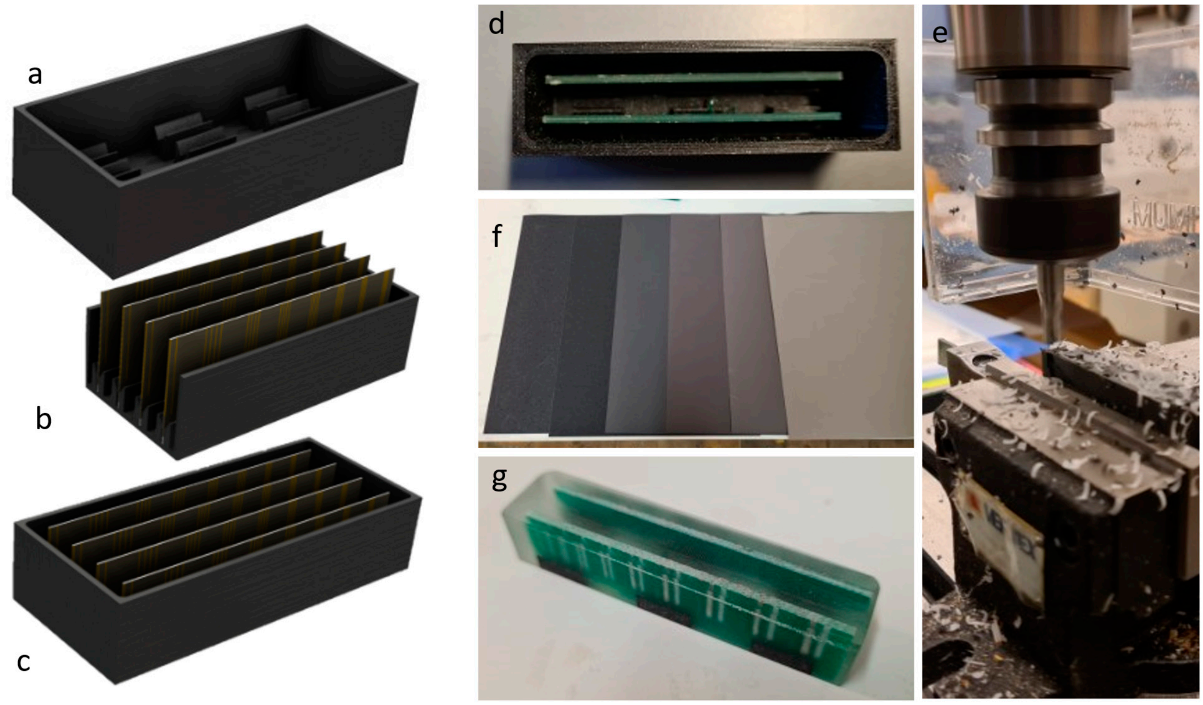

Fig. S4. Sequential representation of the PCB cross sectioning. (a) Depicts an empty 3D-printed mold. (b) Shows a cross-section of the 3D-printed mold with flexible PCBs inserted. (c) Presents the mold after trimming the PCBs. (d) Displays the 3D-printed mold securely holding cut FR-4 PCBs. (e) Reveals the epoxy cast within the 3D-printed mold with the FR-4 PCB enclosed. (f) Highlights a range of sandpapers, progressively increasing in fineness for surface refinement. (g) Illustrates the epoxy casting with the FR-4 PCB fully encased being milled to reveal the cross-sectional view.

5. Peel Testing

To prepare the test samples, two circular blades are mounted with a 10 mm spacing (**Fig S5. A**) to cut exact 10 mm × 80 mm strips out of the 5mm thick PDMS which was cast on top of each of the five individual substrate surfaces. Each PDMS strip is bonded with a microscope slide on a segment that is 20 mm long. The PDMS surface is plasma cleaned for 30 s and bonded with the glass slide. It is then heat treated at 80 °C for 20 minutes. The binding surface area is 20 × 10 mm. The samples were mounted on a linear rail (**Fig. 3a**) with a 3D printed fixture (**Fig. 3e**) to hold them in the bottom clamp of a universal testing machine (MTC 100, IDM Test, Spain). This fixture features a flange to hold the slide on one side and a screw clamp to keep it in place during testing. The end of the PDMS strip is held firmly in the top clamp at a fixed height of 50 mm (**Fig. 3d**) to perform a 90° peel test. As the upper jaw (**Fig. 3e**) translates upwards, the linear rail **(Fig. 3c)** slides horizontally, so when the sample starts peeling away it is always situated perfectly under the clamping jaws and the peeling angle remains constant.

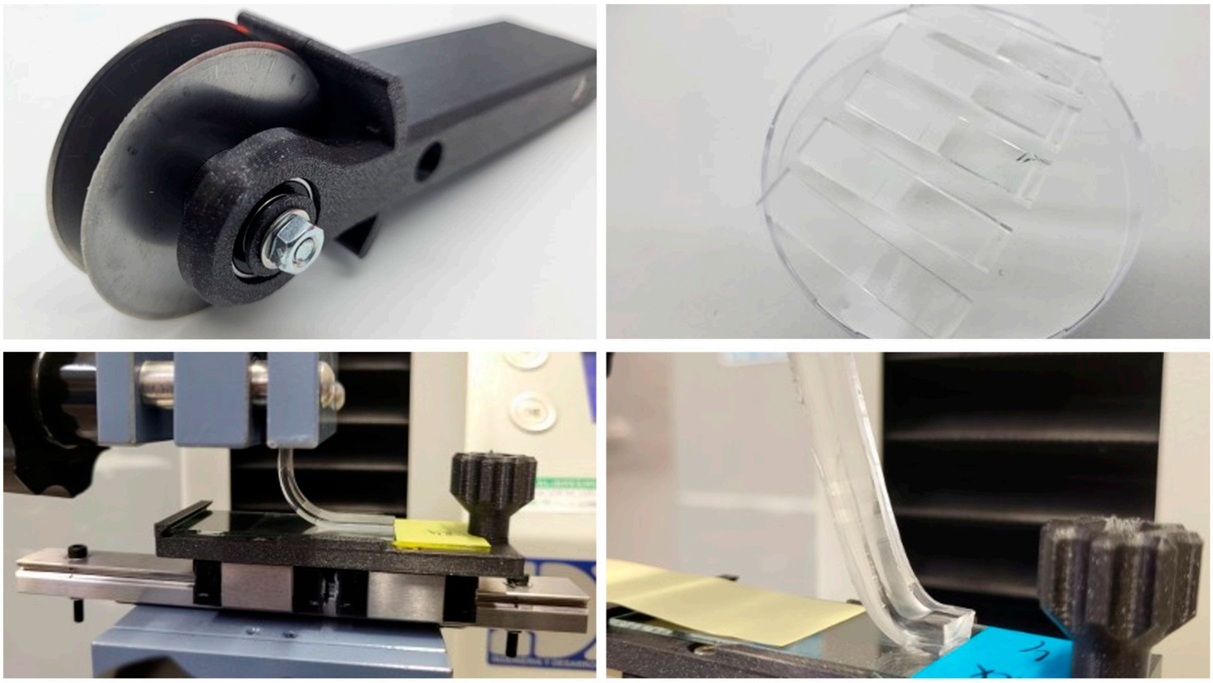

Fig. S5. (a) 3D-printed custom double circular blade used to cut exact 20mm wide PDMS strips. (b) Several PDMS strips with different surface replications prepared for testing. (c) 90° Peel test ready to start. (d) close up of stretching and pulling off the sample during the 90° pull test.

6. Biocompatibility

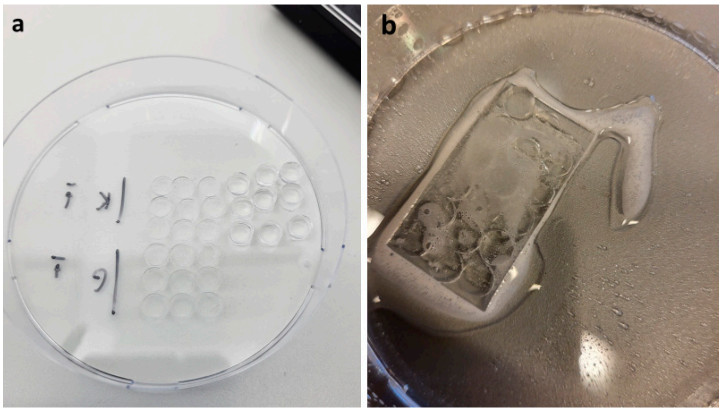

Fig. S6. (a) PDMS samples punched out from a cast covering several surfaces, selected for cytotoxicity testing. (b) Several samples covered in RainX water repellent spray to prepare for testing.

RESULTS

1. Cross sectional PCB Feature Analysis

1.1. Track Geometry

1.2. Track Spacing

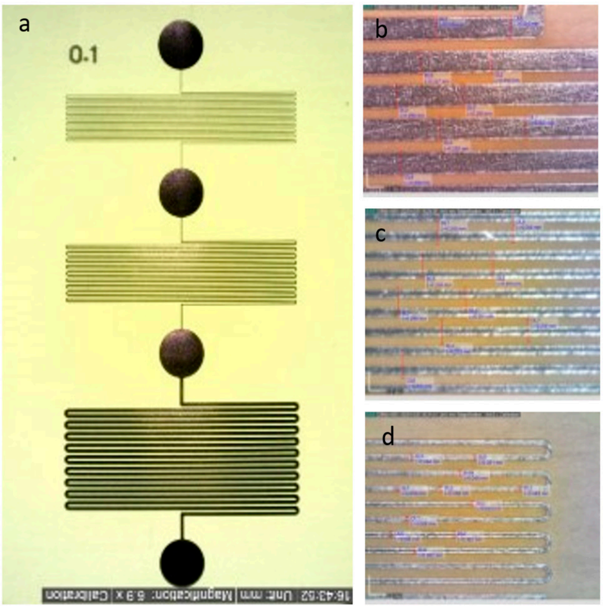

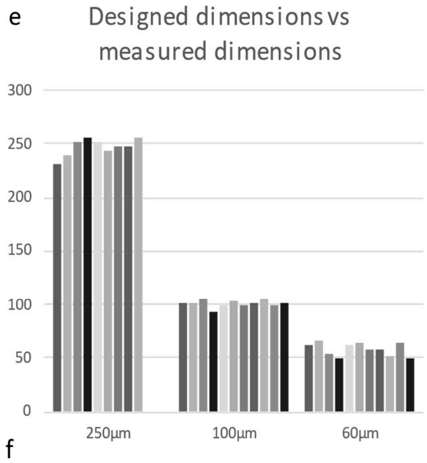

f

	250µm	100µm	60µm
**Avg**	247	101	58
**Std**	9	4	6
**% dev**	3.4	1.5	2.5

Figure S7. (a) Serpentine channels flowing into each other. (b) Random measurements on a 250 µm serpentine track. (c) Random measurements on a 100 µm serpentine track. (d) random measurements on a 60µm serpentine track. (e) Bar chart of the designed channel width compared to the measured dimensions. (f) Overview of the average, standard deviation and relative deviation of the measurements of the 250 µm, 100 µm and 60 µm tracks

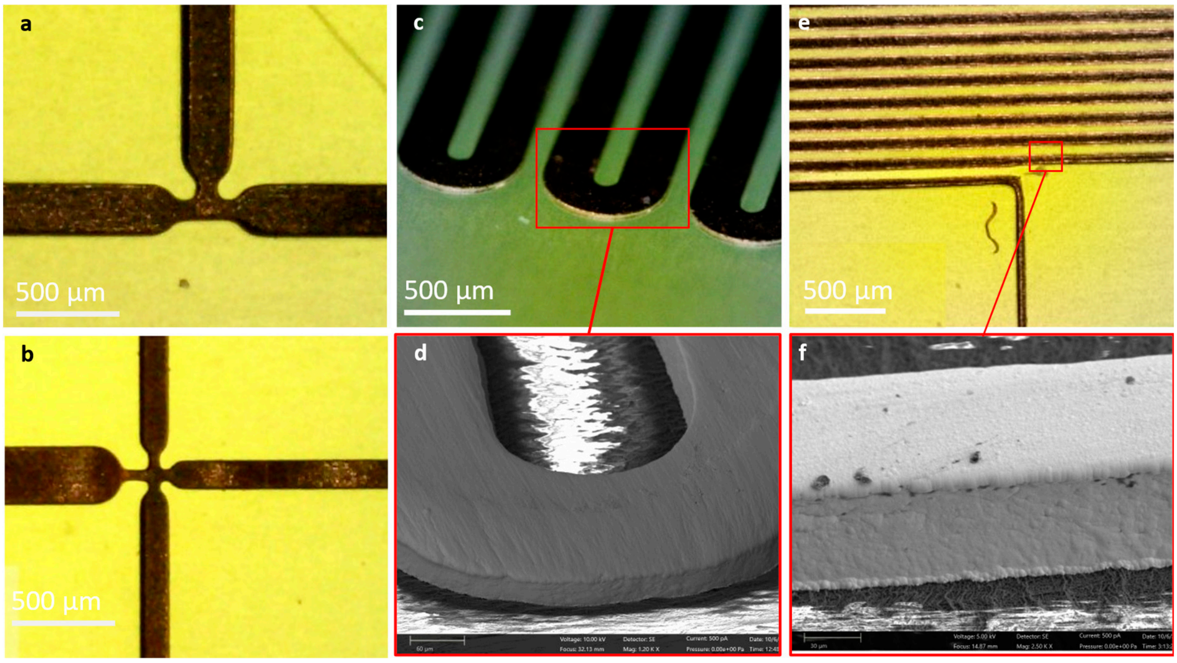

Fig. S8. Microscopic examination of flexible PCB features. (a) Close-up top view microscope image reveals a T-shaped droplet generator pattern. (b) Microscope image from a top view showcases a cross-shaped droplet generator. (c) Microscope image offers a perspective on the serpentine bends. (d) Close-up electron microscope image provides intricate details of one of these bends. (e) Shows serpentine tracks, minimized to their smallest possible dimensions. (f) Close-up scanning electron microscope image offers a detailed view of a specific section of a track.

2. Peel Test

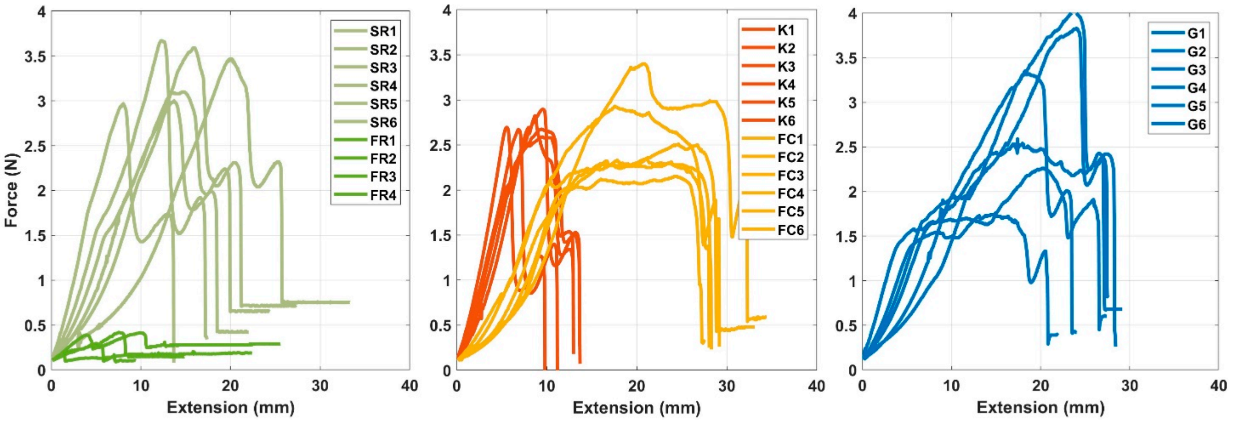

Fig. S9. Overview of the graphs generated during the pull test with the first graph showing the results of Solder Resist (SR1 through SR6) and rough FR-4 (FR1 through FR4), the second graph showing Kapton tape/Coverlay (K1 through K6) and Flexible Core (FC1 through FC6) surface substrate and the last graph showing PDMS cast on the glass, then rebondedn to a microscope slide (G1 through G6)

3. MF Devices – benchmark testing

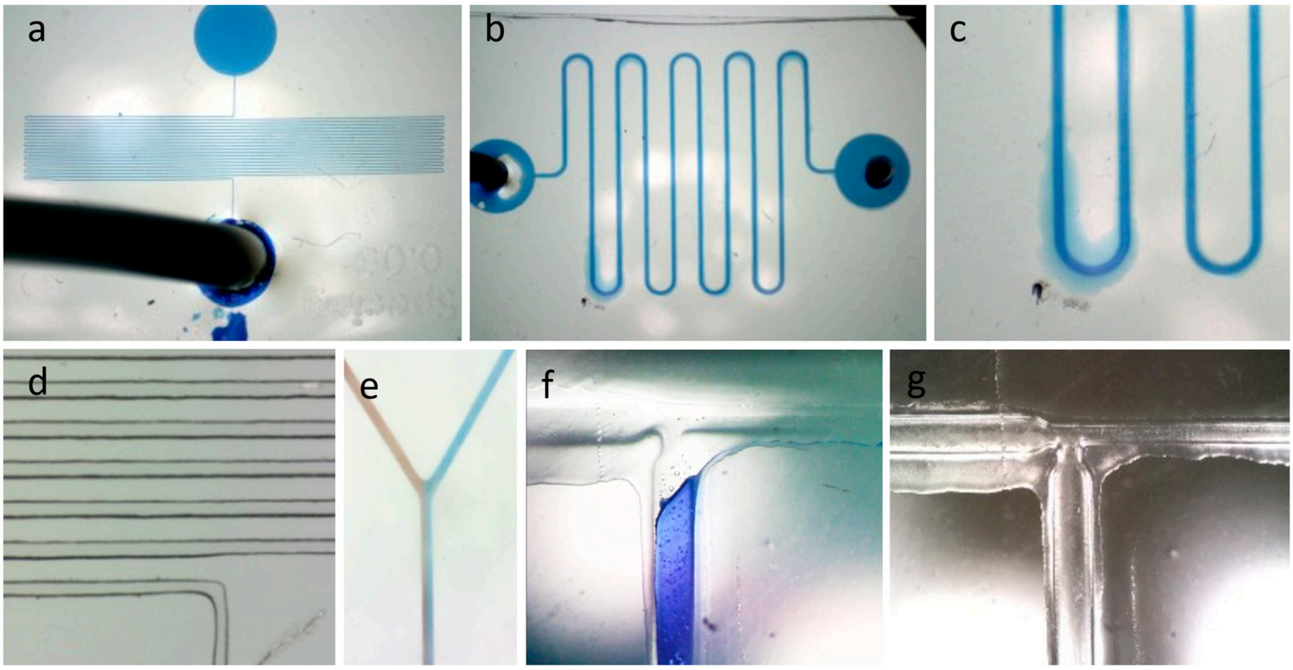

Fig. S10. microcrofluidic Channel Comparisons and Droplet Generators. (a) Serpentine channels with a width of 60µm and a spacing of 60µm produced using flexible PCB patterning. (b) Serpentine channel with a width of 400µm and a spacing of 2mm produced using rigid PCB with solder resist, highlighting edge feathering, particularly around corners. (c) Close-up view of edge feathering in channels. (d) Close-up of channels produced with an exposed flexible PCB. (e) Color mixer demonstrating the merging of two dyes into a single flow using flexible PCB. (f) T-junction droplet generator created with a solder resist-covered FR-4 PCB, with deviations from intended droplet generation locations. (g) T-junction droplet generator produced with solder resist-covered FR-4 PCB.

4. Biocompatibility

The images captured during this study were obtained using an inverted fluorescence microscope (Zeiss Axio Observer Z.1). The top images (see Figure S9, panels a-b) represent the growth of cells under control conditions. In Figure S9, panel c, the images illustrate cell growth on the bottom of the well plate, which was employed for incubating cells on the mixed substrate composed of gold-coated copper and polyimide core. This growth closely resembles that observed in the control conditions, suggesting minimal interference from the introduced materials.

However, Figure S9, panel d, reveals a noteworthy disparity in growth between the bottom and surface of the mixed sample. Notably, there is significantly more growth at the bottom of the well, which partially obstructs the view of the sample's surface, particularly on the left side of the image.

In Figures S9, panels e-f, the growth on the top surface of a mixed sample treated with Rain-X hydrophobic spray is compared to that of Figures S9, panels g-h, where no such treatment was applied. It is evident that the sample subjected to Rain-X treatment exhibits notably improved growth. This observation suggests that while the sample does not exhibit any signs of toxicity, as evidenced by robust growth at the bottom of the well, the cells used in the experiment display a preference for the Rain-X treated surface for their proliferation.

These findings emphasize the influence of surface properties on cell behavior, as even without signs of toxicity, the treated surface demonstrated enhanced cellular compatibility and facilitated more favorable growth conditions.

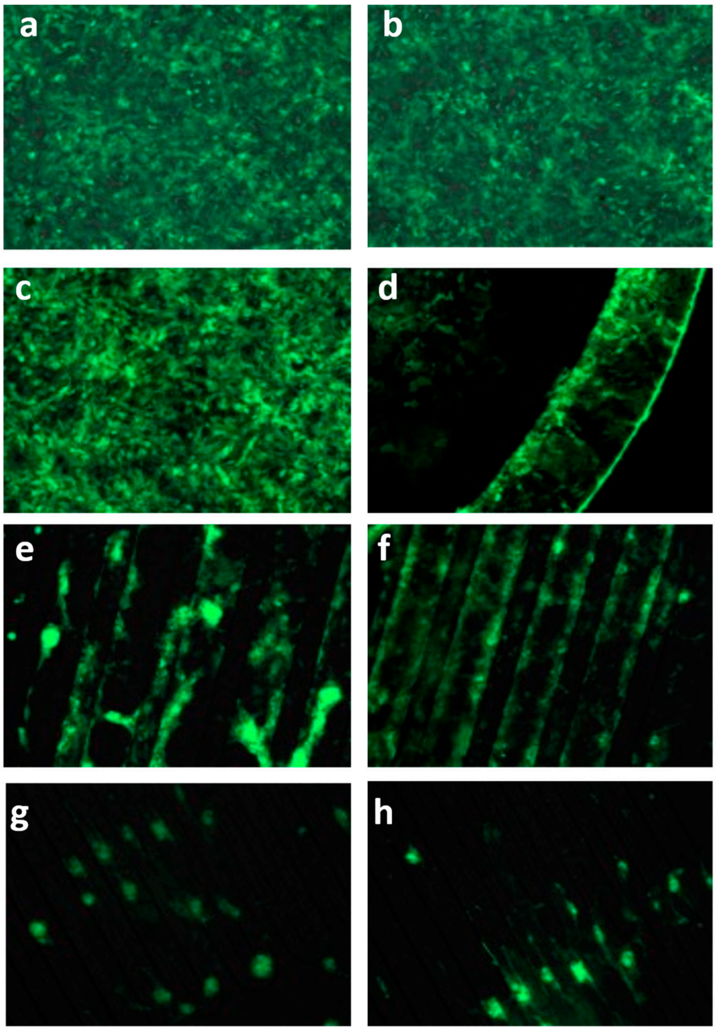

Fig. S11. Cellular growth patterns and the impact of surface treatments. (a-b) Control conditions, (c) Growth at the bottom of the well plate on the mixed substrate of gold-coated copper tracks and polyimide, (d) Comparison of growth between the well bottom and the surface of the mixed sample, (e-f) Growth on the Rain-X treated mixed sample, (g-h) Growth on the mixed sample without treatment.
